# Integrated Metabolomic-Transcriptomic Analysis Reveals Diverse Resource of Functional Ingredients From Persimmon Leaves of Different Varieties

**DOI:** 10.3389/fpls.2022.904208

**Published:** 2022-05-25

**Authors:** Xian-Mei Yu, Jie Wang, Rui Gao, Bang-Chu Gong, Cheng-Xiang Ai

**Affiliations:** ^1^Shandong Institute of Pomology, Tai’an, China; ^2^Research Institute of Subtropical Forestry, Chinese Academy of Forestry, Fuyang, China

**Keywords:** persimmon leaf, metabolomics analysis, transcriptomic analysis, functional nutrients, flavonoids, mineral contents, aroma compounds

## Abstract

Persimmon leaves are used for making persimmon leaf tea or as functional ingredients due to their enrichment in flavonoids, the beneficial mineral contents, and favorable flavors contributed by volatile aroma compounds. The varieties/cultivars had a significant influence on the quality and flavor of persimmon leaf tea. In this study, the integrated metabolomic-transcriptomic analysis was conducted to investigate the potential in flavonoid biosynthesis, mineral absorption, and degradation of aromatic compounds from tender leaves of “*Diospyros kaki.* Heishi” (HS), “*Diospyros kaki* Thunb. Nishimurawase” (NM), and “*Diospyros kaki* Thunb. Taifu” (TF), using rootstock “*Diospyros Lotus* Linn” (DL) as the control. The metabolomic analysis showed that 382, 391, and 368 metabolites were differentially accumulated in the comparison of DL vs. HS, DL vs. NM, and DL vs. TF, respectively, and 229 common metabolites were obtained by comparative analysis. By RNA sequencing, 182,008 unigenes with 652 bp of mean length were annotated and 2,598, 3,503, and 3,333 differentially expressed genes (DEGs) were detected from the comparison of DL vs. HS, DL vs. NM, and DL vs. TF, respectively. After the Gene Ontology (GO) and the Kyoto Encyclopedia of Genes and Genomes (KEGG) enrichment, 6, 6, and 3 DEGs [with | log2(fold change)| ≥ 1 simultaneously in the three comparisons] involved in flavonoid biosynthesis, mineral absorption, and degradation of aromatic compounds, respectively, were selected for quantitative reverse transcription-polymerase chain reaction (qRT-PCR) validation and the consistent trends of the relative expression level of each DEG with RNA sequencing (RNA-seq) data were observed. Based on the transcriptomic analysis and qRT-PCR validation, it was observed that the leaves of HS, NM, and TF had the greatest level of mineral absorption, flavonoid biosynthesis, and degradation of aromatic compounds, respectively. In addition, a positive correlation between the 15 DEGs and their metabolites was observed by the conjoint analysis. Thus, the tender leaves of HS, NM, and TF could be recommended for the production of persimmon leaf tea rich in mineral elements, flavonoid, and aroma compounds, respectively.

## Introduction

Persimmon (*Diospyros kaki* L.) is originated in China and is cultivated in warm regions (Ashtiani et al., 2016). Persimmon is cherished for possessing high nutritional and nutraceutical values, due to its high nutrient content and unique flavor based on its primary and secondary metabolites (Giordani et al., 2011; Zhang et al., 2018). Persimmon leaves and their extracts have been reported to be rich in natural antioxidant compounds, especially flavonoids and, therefore, possess the antioxidant capacity (Liu et al., 2012; Martínez-Las Heras et al., 2014, 2016). Due to their enrichment in flavonoids, persimmon leaves have been used as traditional Chinese medicine for a long time to treat ischemia stroke, internal hemorrhage, atherosclerosis, angina, hypertension, and some other infectious diseases (Chang et al., 2019). On the other hand, persimmon leaves have also been used as an ingredient of herbal beverages for centuries in eastern Asia because they are beneficial to human health and their various components have health-promoting effects (Chen et al., 2009; Yaqub et al., 2016; Hossain et al., 2018; Kim et al., 2020). Persimmon leaf tea has become increasingly popular in Asian countries, such as China, Japan, and South Korea.

Minerals, such as Fe, Cu, Cr, Mn, and Zn, are essential and beneficial elements for humans. The mineral contents in herbal teas are extraordinary, attracting consumers not only from the nutritional viewpoint, but also to assess the quality and evaluate the potential benefit from their consumption (Bhat et al., 2010). Mineral elements of food, especially in agricultural products and their corresponding soil, are stable, which can be used as the signature of geographical origin of the product (Zhao et al., 2017b; Li et al., 2018; Tardugno et al., 2021). Meanwhile, the factors, such as geographical origin, variety, climate conditions, harvest season or growth stage, and their interactions had a significant influence on mineral contents and quality of tea leaves (Jia et al., 2016; Zhao et al., 2017a).

Tea is popular worldwide partly due to its favorable flavor and taste (Yang et al., 2013). Aromatic aroma compounds, including volatile compounds and non-volatile compounds, contribute to tea flavor. Volatile aroma compounds are representative and specialized metabolites in tea leaves, which contribute to the aroma property of tea flavor and determine the final quality and value of tea products (Naveed et al., 2018; Jiang et al., 2019; Wang et al., 2019).

Persimmon cultivars are classified into the four groups: pollination-constant non-astringent (PCNA), pollination variant non-astringent (PVNA), pollination-constant astringent (PCA), and pollination variant astringent (PVA). PVNA, PCA, and PVA were also categorized as non-PCNA types (Akagi et al., 2011; Yang et al., 2016). Variety/cultivar was one of the important impacting factors on the compositions and contents of flavonoids in persimmon leaves, which were significantly correlated with their antioxidant activities and the leaves from PCA persimmons had higher levels of total flavonoid and, thus, had better antioxidant effects, followed by PVNA persimmons and PCNA persimmons; the PVA variety had the lowest amount of total flavonoid (Chang et al., 2019).

Persimmon leaf tea has been used as a pleasant, functional beverage and an effective, traditional herbal remedy for a long time (Chen et al., 2009; Chang et al., 2019), but how to increase the content of functional compounds and improve the quality and flavor of persimmon leaf tea by screening the varieties/cultivars rich in flavonoid, mineral, or aroma compounds are largely unexplored. In recent years, integrative metabolomic-transcriptomic analysis has been extensively applied to reveal the relationship between the contents of secondary metabolites and their corresponding differentially expressed genes (DEGs; Zhou et al., 2019, 2022; Wan et al., 2020, 2021; Zhang et al., 2020; Qi et al., 2021; Wang et al., 2021).

In this study, we conducted the integrated metabolomic-transcriptomic analysis to investigate the flavonoid biosynthesis, mineral absorption, and degradation of aromatic compounds from tender leaves of different persimmon varieties, including PCA type “*Diospyros kaki.* Heishi” (HS), PVNA type “*Diospyros kaki* Thunb. Nishimurawase” (NM), and “*Diospyros kaki* Thunb. Taifu” (TF), using the rootstock “*Diospyros Lotus* Linn” (DL) as the control. We hope that this study would provide insight into the functional ingredients from persimmon leaves of different types or varieties/cultivars and the results would facilitate the selection of choice varieties/cultivars of persimmon for optional persimmon leaf tea with particular functional ingredients.

## Materials and Methods

### Plant Materials

The persimmon varieties of rootstock “*Diospyros Lotus* Linn” (DL), PCA type “*Diospyros kaki.* Heishi” (HS), PVNA type “*Diospyros kaki* Thunb. Nishimurawase” (NM), and “*Diospyros kaki* Thunb. Taifu” (TF) used in this study were grown in the persimmon test base of Shandong Institute of Pomology in Tai’an, China under natural conditions. On a sunny morning, 20 g of tender leaves were sampled from persimmon trees and three trees of each persimmon variety were used for sampling as three biological replicates.

### Metabolomic Profiling and Metabolite Data Analysis

The sample preparation, extract analysis, metabolite identification, and quantification were performed at Wuhan Metware Biotechnology Co., Ltd., WuHan, China following^1^ the standard procedures, which were previously described by Yuan et al. (2018), Cao et al. (2019), and Zhang et al. (2019). Metabolite data analysis was carried out using the Analyst version 1.6.1 software (AB SCIEX, ON, Canada).

### Ribonucleic Acid Extraction, Quantification, and Sequencing

Twelve complementary DNA (cDNA) libraries of four samples and three replicates were constructed for transcriptome sequencing. The extraction and quantification of total RNA, construction of cDNA libraries, and sequencing were completed at Wuhan Metware Biotechnology Co., Ltd. (see text footnote 1). The libraries were sequenced on the Illumina HiSeq™ 4000 platform and the 150 bp paired-end reads were generated.

### Transcriptome Data Analysis

Raw reads were processed with FastQC^2^ and clean reads were obtained by removing the adapters and low-quality sequences. *De novo* assembly of the clean reads was performed using Trinity version 2.5.1^3^ (Grabherr et al., 2011; Liu et al., 2013). Quantification of gene expression level was carried out using the featureCounts version 1.5.0-P3 (Liao et al., 2014) based on the expected number of fragments per kilobase of transcript sequence per millions of base pairs sequenced (FPKM) method (Trapnell et al., 2010). Differential expression analysis between the two compared groups (three biological replicates per group) was performed using the DESeq2 R package (version 1.20.0) (Love et al., 2014) with adjusted *P*-values. The differentially expressed genes (DEGs) were identified with the following parameters: adjusted *P*-value < 0.05 and | log2(fold change)| ≥ 1. The Gene Ontology (GO) and the Kyoto Encyclopedia of Genes and Genomes (KEGG) enrichment of DEGs were implemented by the GOseq R package (version 2.28.0) (Young et al., 2010) and KOBAS software (version 2.0) (Mao et al., 2005; Wu et al., 2006), respectively.

### Quantitative Reverse Transcription-Polymerase Chain Reaction Validation of the Selected Differentially Expressed Genes

To validate the accuracy of DEGs obtained from the assembled datasets and profiling of gene expression *via* RNA sequencing (RNA-seq), quantitative reverse transcription-polymerase chain reaction (qRT-PCR) was carried out for the selected 15 DEGs, with the parameters of | log2(fold change)| ≥ 1 and *P*-value < 0.05 simultaneously in the three comparisons, using the primers shown in Table 1. The total RNAs used for RNA-seq were also used as the template for the qRT-PCR validation, glyceraldehyde-3-phosphate dehydrogenase (*GAPDH*) was used as the reference gene, and three biological replicates were analyzed.

**TABLE 1:** The quantitative reverse transcription-polymerase chain reaction (qRT-PCR) primers and their sequences for the 15 selected differentially expressed genes (DEGs).

Gene ID	Predicted function	KEGG pathway	Primer sequences (5′-3′)
*DN44356_c5_g1*	Flavonoid biosynthesis	ko00941	F: CGCCATCAACTCCCTGAATAG
			R: GGAGCCAAGGAAAGGCTAAA
*DN46460_c0_g2*	Flavonoid biosynthesis	ko00941	F: GGGATAGGATGAGATGCCTTTG
			R: CCGACGACTCCATTGATGATT
*DN63124_c2_g2*	Isoflavonoid biosynthesis	ko00943	F: CAAAGGCAACTGTCGTTTCTG
			R: GCAGGCGCATCGTCTAATA
*DN27169_c0_g1*	Isoflavonoid biosynthesis	ko00943	F: CTACGCATGTGGAGGTTCAA
			R: GAGGTGAAGCAGTCGACAAT
*DN63124_c2_g1*	Isoflavonoid biosynthesis	ko00943	F: GCTTCTCGGTTTGGGTCTATT
			R: GCCAAGAGGACACTAGAGATTG
*DN50820_c3_g2*	Isoflavonoid biosynthesis	ko00943	F: ACCACCTCCTCCTTTCTCA
			R: TCTCTCTCTCTCTCTCTCTCTCT
*DN41367_c2_g1*	Mineral absorption	ko04978	F: AGCTAAGAAGAGTGGGCAAAG
			R: GAAGGGAACCAAGACCAGATT
*DN25744_c0_g1*	Mineral absorption	ko04978	F: CTTTCAACAGCCGCCTTATTC
			R: TACACTCAAGCCCACACATAC
*DN35062_c3_g1*	Mineral absorption	ko04978	F: TCTATCGCTCGCCAGAAGTA
			R: TCGGAAACAGAGGATCGAAATAAG
*DN45693_c0_g2*	Mineral absorption	ko04978	F: GCTTGACCTCTTTCCCTTCTT
			R: CCAACCCTTTACCAGTCTTTCT
*DN51444_c1_g2*	Mineral absorption	ko04978	F: CGGAGCGAGACTGGATAATAAG
			R: GCTGGAGCTGACCACAAATA
*DN51444_c1_g1*	Mineral absorption	ko04978	F: TAGGTCAACAGTGCAACATAGAG
			R: ATGGGATGGATGTCAAGTAACC
*DN62817_c3_g2*	Degradation of aromatic compounds	ko01220	F: ACAGCAGGCAAACCCATAA
			R: CTGGAGGAGCTACTTCGATTTC
*DN35893_c0_g1*	Degradation of aromatic compounds	ko01220	F: AAGACCAATCTGTGCGAGAAG
			R: GGTGGACGTGTTGAGGAAAT
*DN62817_c3_g3*	Degradation of aromatic compounds	ko01220	F: CTGAGTTTGGACTCGCTTCA
			R: GGACTGGAATATCAGACTTGGG
GAPDH	Reference gene		F: AGCTCTTCCACCTCTCCAGT
			R: TGCTAGCTGCACAACCAACT

### Conjoint Analysis of Metabolome and Transcriptome Data

For the conjoint analysis, the mean of metabolome data and transcriptome data was calculated and then the Pearson correlation (*r*) between metabolites and transcripts represented by network diagrams was calculated (Cho et al., 2016). Metabolome and transcriptome relationships were visualized using Cytoscape version 3.6.1 (Su et al., 2014).

## Results

### Metabolome Profiles

**FIGURE 1:**
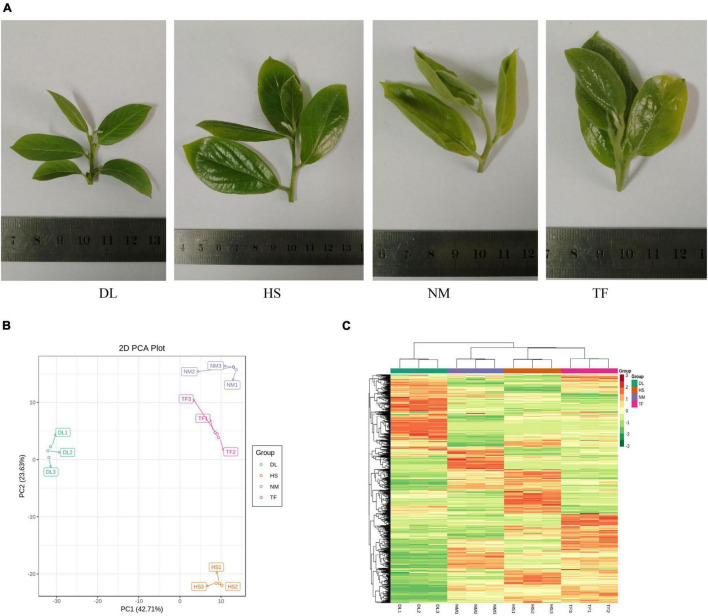
The metabolome profiles of the four samples of *Diospyros Lotus* Linn (DL), *Diospyros kaki.* Heishi (HS), *Diospyros kaki* Thunb. Nishimurawase (NM), and *Diospyros kaki* Thunb. Taifu (TF). **(A)** The samples in this study. **(B)** The principal component analysis (PCA) plot. **(C)** Heatmap of the metabolites from the four samples.

After profiling the metabolome of the four samples (Figure 1A) and three replicates using the widely targeted metabolomic approach, we detected 868 compounds clustered into 22 classes. The principal component analysis (PCA) of the four samples based on the metabolites showed that all the biological replicates clustered together (Figure 1B), indicating the reliability of the metabolome data. It was observed that the four samples were clustered into the two main groups in the heatmap (Figure 1C), suggesting that the metabolite profiles of DL were obviously distinct from those of HS, NM, and TF.

### Identification of the Differentially Accumulated Metabolites

**FIGURE 2:**
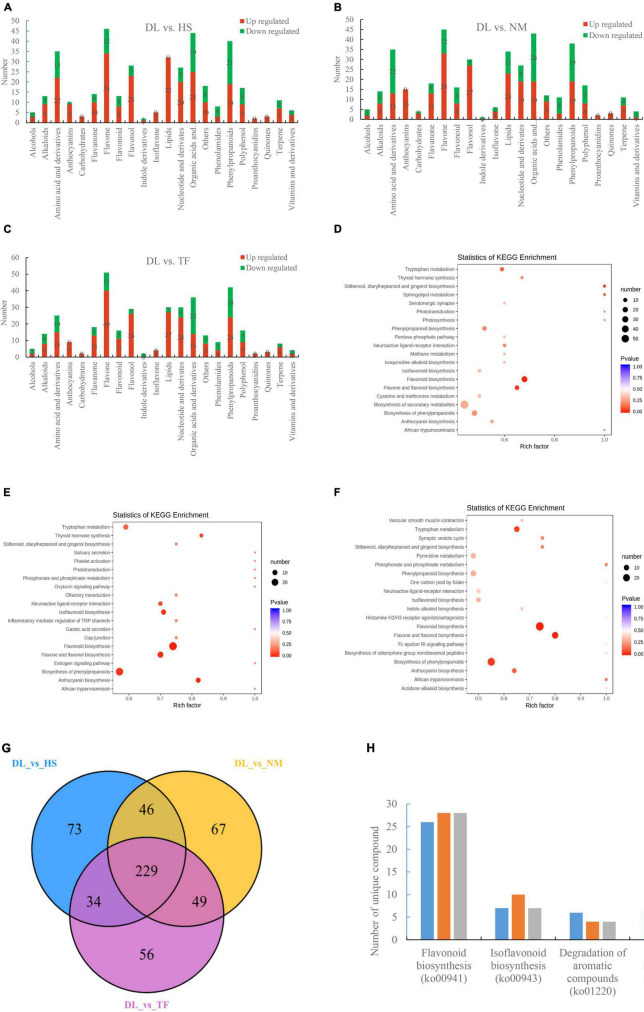
Identification and functional characterization of the differentially accumulated metabolites (DAMs) between the groups. **(A–C)** The metabolites accumulated in the comparison of DL vs. HS, DL vs. NM, and DL vs. TF, respectively. **(D–F)** The top enriched Kyoto Encyclopedia of Genes and Genomes (KEGG) terms of the DAMs from the comparison of DL vs. HS, DL vs. NM, and DL vs. TF, respectively. **(G)** Venn diagram depicting the shared and specific metabolites between the compared groups. **(H)** The unique DAMs related to flavor accumulated in the three compared groups.

The differentially accumulated metabolites (DAMs) between the compared groups were determined based on the variable importance in projection (VIP) ≥1 and fold change ≥2 or fold change ≤0.5 (Yuan et al., 2018). A total of 382, 391, and 368 metabolites were differentially accumulated in the comparison of DL vs. HS, DL vs. NM, and DL vs. TF, respectively (Figures 2A–C). The top enriched KEGG terms of the DAMs detected for the three compared groups were metabolic pathways, biosynthesis of secondary metabolites, flavonoid biosynthesis, biosynthesis of plant secondary metabolites, flavone and flavonol biosynthesis, and isoflavonoid biosynthesis (Figures 2D–F). Comparative analysis of the three compared groups resolved to 229 common metabolites (Figure 2G). The unique DAMs related to the objective flavor of persimmon leaves were detected from the three compared groups, which are shown in Figure 2H.

### Transcriptome Profiles

**FIGURE 3:**
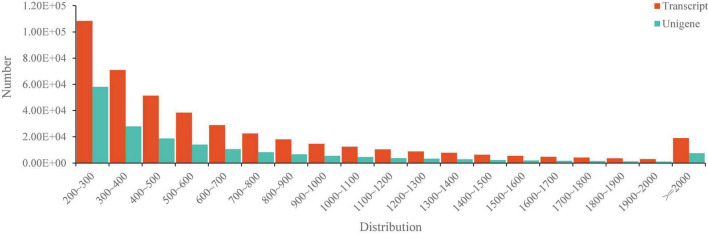
The length distribution of the transcriptome sequencing data.

By construction of the 12 libraries and RNA sequencing, 779,759 million raw reads were obtained and the dataset of raw reads was deposited in the National Center for Biotechnology Information (NCBI) database with the accession number PRJNA646845^4^. After clean-up and quality filtering, 770,810 million clean reads containing 115.18 Gb clean bases were obtained and the bases scoring Q20 (base quality more than 20) and Q30 (base quality more than 30) were 97.48 and 93.06%, respectively (Table 2). By using the trinity assembling program, 439,550 transcripts with 693 bp of mean length were obtained and 182,008 unigenes with 652 bp of mean length were annotated. The transcripts and unigenes of <500 bp accounted 52.51 and 57.56%, respectively (Figure 3), indicating the high quality of RNA sequencing data.

**TABLE 2:** The quality control statistics of the transcriptome sequencing data.

Sample	Raw reads	Clean reads (percent %)	Clean base	Error rate	Q20*	Q30*
DL1	75,737,150	74767370 (98.72%)	11.17G	0.03%	97.49%	93.11%
DL2	57,966,176	57367986 (98.97%)	8.56G	0.03%	97.90%	94.13%
DL3	68,325,480	67068192 (98.16%)	10.01G	0.03%	97.40%	92.86%
HS1	54,569,578	53941216 (98.85%)	8.07G	0.03%	97.18%	92.30%
HS2	61,770,486	61003740 (98.76%)	9.12G	0.03%	97.29%	92.62%
HS3	69,508,062	68734464 (98.89%)	10.27G	0.03%	97.39%	92.86%
NM1	52,626,190	52115670 (99.03%)	7.79G	0.03%	97.76%	93.83%
NM2	63,795,416	63117626 (98.94%)	9.44G	0.03%	97.36%	92.73%
NM3	51,492,512	50902372 (98.85%)	7.60G	0.03%	97.57%	93.42%
TF1	81,688,742	80789064 (98.90%)	12.08G	0.03%	97.33%	92.67%
TF2	83,930,758	83095536 (99.00%)	12.41G	0.03%	97.46%	92.96%
TF3	58,348,814	57906760 (99.24%)	8.66G	0.03%	97.61%	93.19%

*Q20 and Q30 are the percentage of bases with a Phred value greater than 20 and 30 as a percentage of total bases, respectively.

**FIGURE 4:**
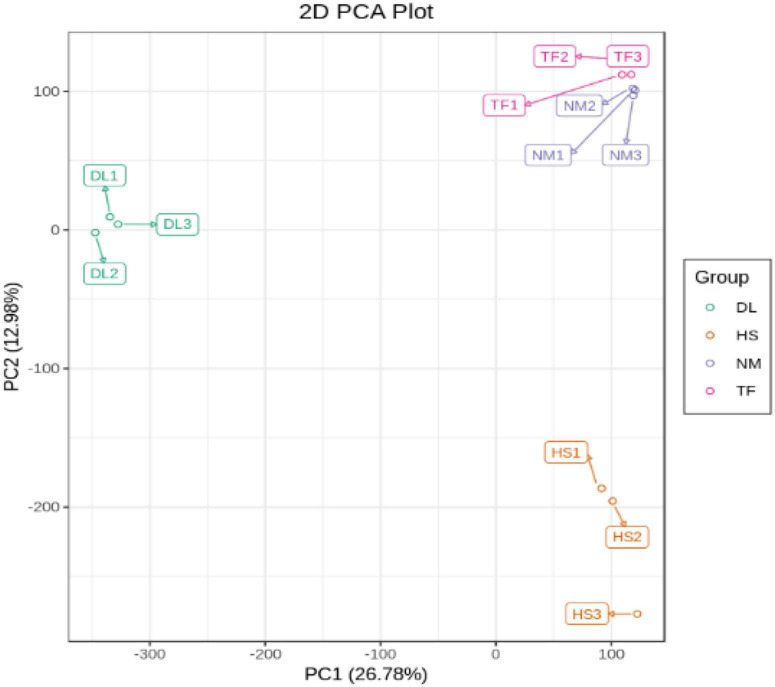
The PCA diagram based on the FPKM values of the transcriptome sequencing data.

The principal component analysis (PCA) of the samples based on the FPKM values showed that all the biological replicates clustered together, indicating the reliability of the sequencing data (Figure 4).

### Analysis of Differentially Expressed Genes

**FIGURE 5:**
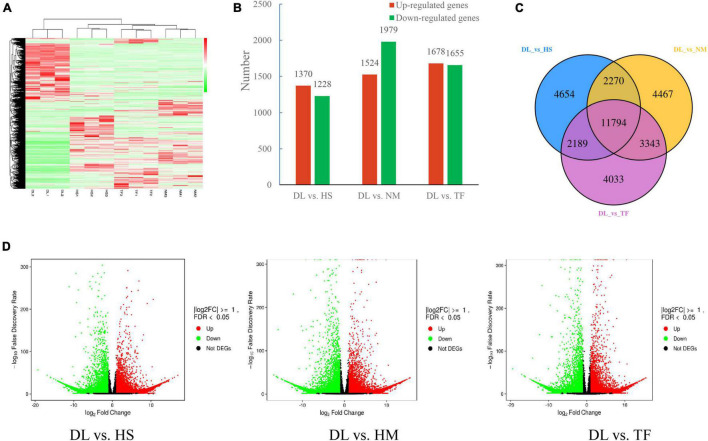
Differentially expressed genes (DEGs) in the three comparisons. **(A)** Heatmap of all the DEGs from the four samples. **(B)** Upregulated/downregulated DEGs in the three comparisons. **(C)** Venn diagrams of DEGs in the three comparisons. **(D)** Volcano plots of the DEGs from the comparison of DL vs. HS, DL vs. HM, and DL vs. TF, respectively, red/green dots indicate upregulated/downregulated expression of genes, respectively, and black dots indicate no difference.

With the filter criteria of | log2(fold change)| ≥ 1 and *P*-value < 0.05, 2,598, 3,503, and 3,333 DEGs were detected from the comparisons of DL vs. HS, DL vs. NM, and DL vs. TF, respectively, of which 1,370, 1,524, and 1,678 genes were upregulated and 1,228, 1,979, and 1,655 genes were downregulated in the comparison of DL vs. HS, DL vs. NM, and DL vs. TF, respectively (Figures 5B,D). Based on the DEGs, the four samples were clustered into the two main groups in the heatmap (Figure 5A), which was consistent with the result of metabolome analysis, indicating the high reliability of the transcriptome sequencing data. Comparative analysis of the three comparisons was resolved to 11,794 common DEGs, which were shown in the Venn diagram (Figure 5C).

**FIGURE 6:**
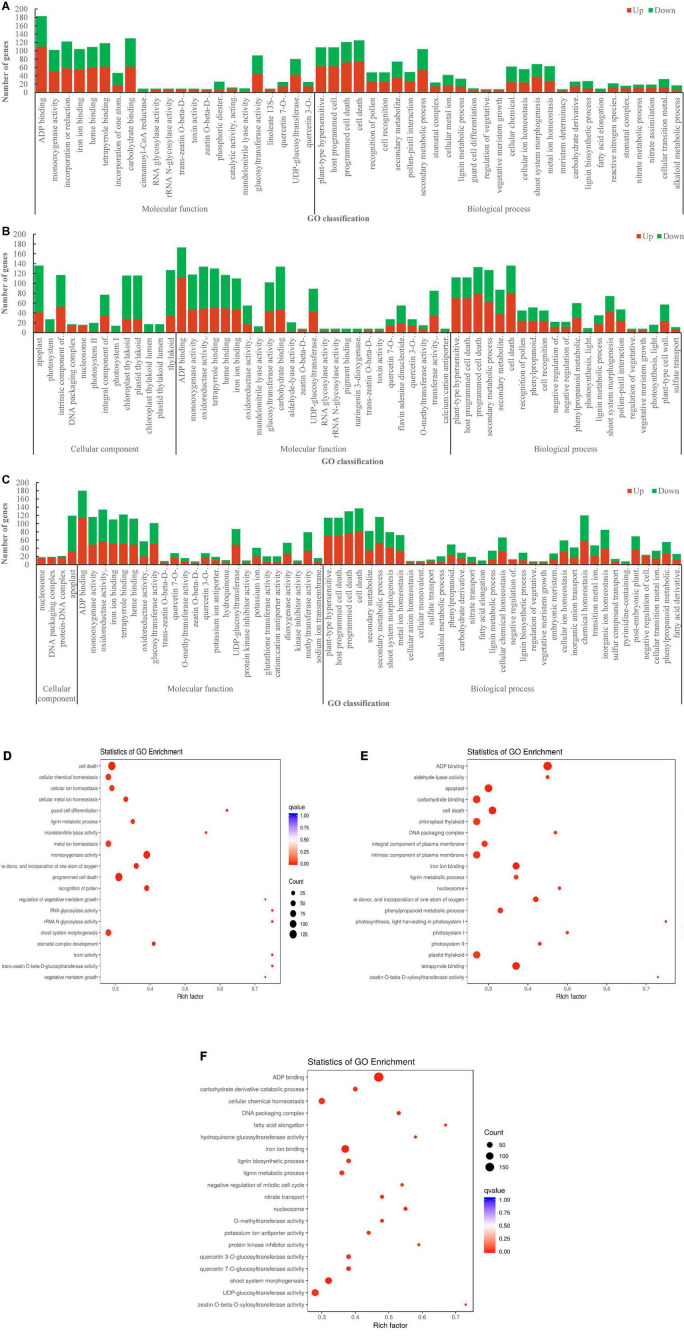
The Gene Ontology (GO) enrichment of DEGs from the three comparisons. **(A)** DL vs. HS. **(B)** DL vs. NM. **(C)** DL vs. TF. **(D–F)** The top enriched GO terms of the DEGs from the comparison of DL vs. HS, DL vs. NM, and DL vs. TF, respectively.

By the Gene Ontology (GO) analysis, the 2,598 DEGs from the comparison of DL vs. HS were enriched into 2 classes and 51 subclasses, of which 1,244 (655 upregulated and 589 downregulated) and 1,354 (715 upregulated and 639 downregulated) DEGs were annotated with the GO terms related to “molecular function” and “biological process” (Figure 6A). The 3,503 DEGs from the comparison of DL vs. NM were enriched into 3 classes and 59 subclasses, of which 817 (253 upregulated and 564 downregulated), 1,469 (623 upregulated and 846 downregulated), and 1,217 (648 upregulated and 569 downregulated) were annotated with the GO terms related to “cellular component,” “molecular function,” and “biological process,” respectively (Figure 6B). The 3,333 DEGs from the comparison of DL vs. TF were enriched into 3 classes and 63 subclasses, of which 177 (85 upregulated and 92 downregulated), 1,387 (675 upregulated and 712 downregulated), and 1,769 (918 upregulated and 851 downregulated) were annotated with the GO terms related to “cellular component,” “molecular function,” and “biological process,” respectively (Figure 6C). The top enriched GO terms of the DEGs from the three comparisons were ADP binding, cell death, programmed cell death, iron ion binding, carbohydrate-binding, secondary metabolic process, and plant-type hypersensitive response, etc. (Figures 6D–F).

**FIGURE 7:**
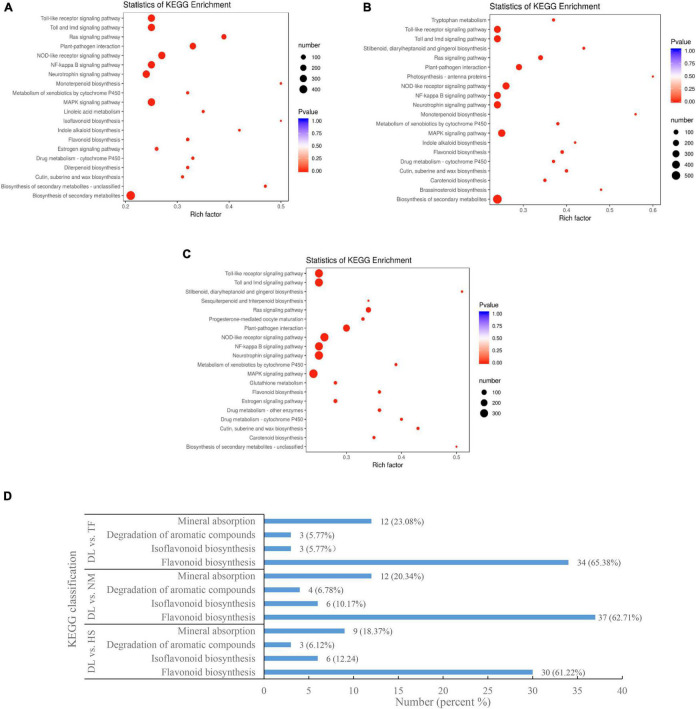
The KEGG enrichment of the DEGs from the three comparisons. **(A–C)** The top enriched KEGG terms from the comparison of DL vs. HS, DL vs. NM, and DL vs. TF, respectively. **(D)** The KEGG of DEGs related to flavonoid biosynthesis, degradation of aromatic compounds, and mineral absorption.

In the Kyoto Encyclopedia of Genes and Genomes (KEGG) pathway enrichment analysis, the top enriched KEGG pathways of the DEGs were biosynthesis of secondary metabolites, Toll and IMD signaling pathway, plant–pathogen interaction, Toll-like receptor signaling pathway, isoflavonoid biosynthesis, and flavonoid biosynthesis, etc. (Figures 7A–C). The KEGG-enriched DEGs involved in flavonoid biosynthesis, isoflavonoid biosynthesis, aroma degradation of aromatic compounds, and mineral absorption from the three comparisons are shown in Figure 7D.

### Quantitative Reverse Transcription-Polymerase Chain Reaction Validation of the Detected Differentially Expressed Genes

**FIGURE 8:**
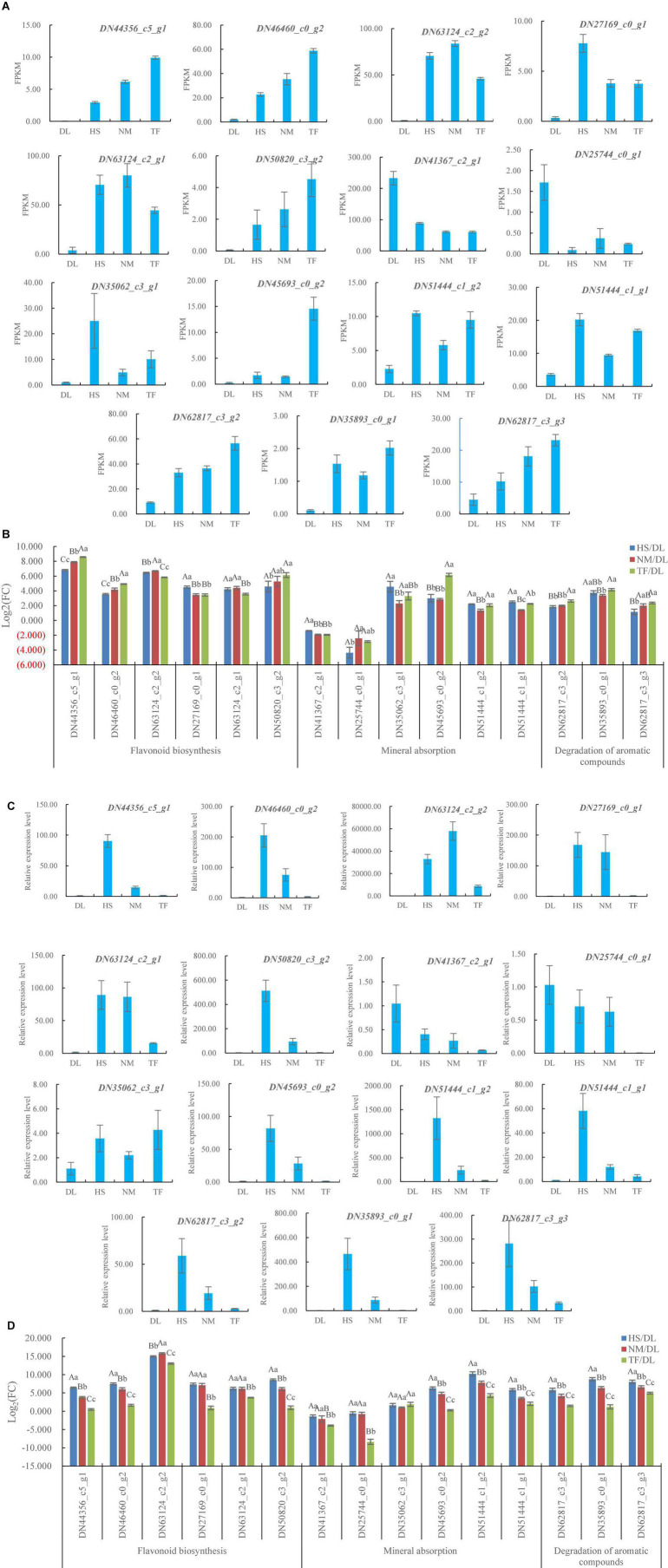
Differentially expressed genes (DEGs) in HS, HM, and TF compared with DL. **(A)** DEGs from RNA sequencing (RNA-seq) data. **(B)** Log2(fold change) of the FPKM for the DEGs from RNA-seq data. **(C)** Relative expression level of the DEGs. **(D)** Log2(fold change) of the relative expression level for the DEGs. Error bars indicate the SD of three biological replicates. The upper case and lower case indicated the significant difference at *P* < 0.01 and *P* < 0.05 level, respectively.

After the GO and the KEGG enrichment, the 15 DEGs related to flavonoid biosynthesis, degradation of aromatic compounds, and mineral absorption, which met with the parameter of | log2(fold change)| ≥ 1 (fold change ≥2 or <0.5) and *P*-value < 0.05 simultaneously in the three comparisons (Figures 8A,B), were selected for qRT-PCR validation (Table 1). Of the 15 DEGs, the FPKM values of 13 DEGs from HS, NM, and TF were more than twice that of DL and the FPKM values of *DN41367_c2_g1* and *DN25744_c0_g1* were 0.5-fold greater than that of DL (Figure 8A), resulting in the | log2(fold change)| of the FPKM for all the 15 DEGs ranging from 1.16 to 8.57 (Figure 8B).

The qRT-PCR results showed that, of the selected 15 genes, the relative expression level of 13 DEGs from HS, NM, and TF was higher than that of DL and the relative expression level of *DN41367_c2_g1* and *DN25744_c0_g1* was less than that of DL (Figure 8C), resulting in the | log2(fold change)| of relative expression level for all the 15 DEGs ranging from 0.26 to 15.79 (Figure 8D). The results were consistent with RNA sequencing data, indicating the reliability of RNA sequencing and qRT-PCR validation.

### Conjoint Analysis of Transcriptome and Metabolome

**FIGURE 9:**
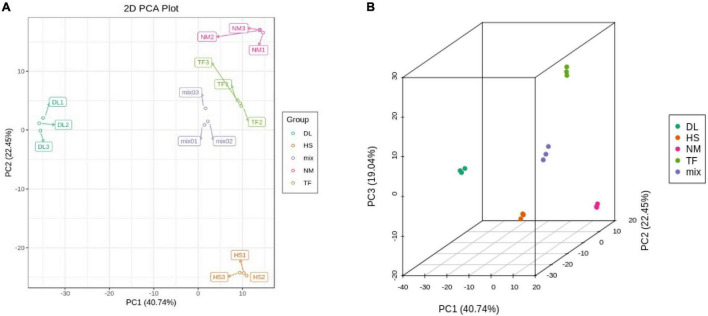
The two-dimensional (2D) PCA plot **(A)** and three-dimensional (3D) PCA plot **(B)** of conjoint analysis.

The principal component analysis (PCA) based on the conjoint analysis of RNA-seq data and metabolome profile showed that the three biological replicates of each samples clustered together in the two-dimensional (2D) PCA plot (Figure 9A) and three-dimensional (3D) PCA plot (Figure 9B), which was consistent with the PCA analysis of RNA-seq data (Figure 4) and metabolome profile (Figure 1B) separately, indicating that the reliability of transcriptome and metabolome profiles.

**FIGURE 10:**
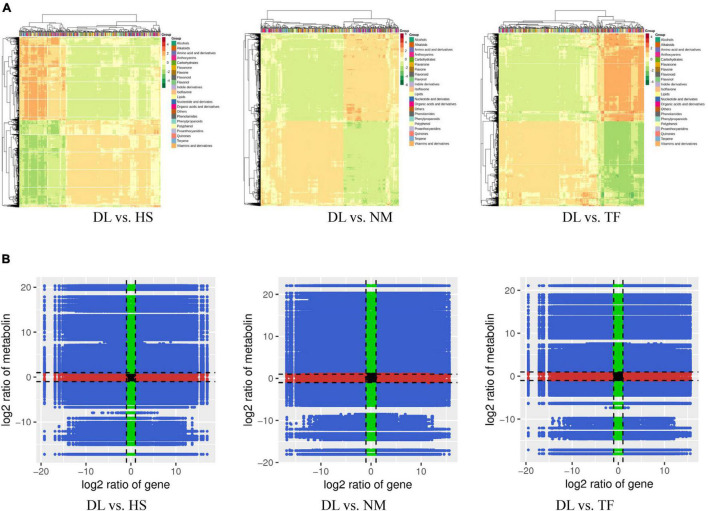
Conjoint analysis of RNA-seq data and metabolome profile from the three comparisons. **(A)** The cor heatmap of the comparison of DL vs. HS, DL vs. NM, and DL vs. TF. **(B)** The nine quadrants diagram of the comparison of DL vs. HS, DL vs. NM, and DL vs. TF. The (night quadrants diagram) were divided into 9 parts (1–9, from left to right, and from up to down in sequence) by the dotted line.

For all the 15 selected DEGs related to flavonoid biosynthesis, degradation of aromatic compounds, and mineral absorption, the Pearson correlation coefficient (PCC) of DEGs was ≥0.8 and all the DEGs were clustered in the cor heatmap, of which the DEGs related to flavonoid biosynthesis were top enriched (Figure 10A). In addition, in the nine quadrants diagram, all the 15 DEGs were located at the quadrant of 3 or 7, which indicate that these DEGs were positively correlated with their metabolites (Figure 10B).

## Discussion

Persimmon leaves can be used for making persimmon leaf tea or as a functional ingredient to add healthy and therapeutic properties (Jo et al., 2003; Sakanaka et al., 2005; Martínez-Las Heras et al., 2016, 2017) due to their possessing high medical values and exhibiting many biological activities, such as neuroprotection, radical scavenging, antithrombotic, antiallergic, and antimutagenic (Kotani et al., 2000; Matsumoto et al., 2002; Sakanaka et al., 2005; Chang et al., 2019), based on their enrichment in flavonoids, which are considered to be the primary active antioxidant components (Liu et al., 2012; Martínez-Las Heras et al., 2014, 2016).

As a functional beverage, the quality and flavor of persimmon leaf tea were primarily determined by the flavonoid contents, mineral contents, and aromatic aroma compounds, which were significantly influenced by the varieties or cultivars of persimmon. It is reported that the leaves from the PCA varieties, such as *D. kaki* cv. Bull Heart, *D. kaki* cv. Diamond Bull Heart, *D. kaki* cv. Aoso, and *D. japonica*, had higher levels of total flavonoid, the leaves from *D. kaki* cv. Amahyakume (PVNA) and *D. kaki* cv. Fuyu (PCNA) contained a lower amount of total flavonoid, and the leaves from the PVA variety *D. kaki* cv. Tonewase contained the lowest amount of total flavonoid (Chang et al., 2019). In this study, we selected PCA persimmon “*Diospyros kaki.* Heishi” (HS), PVNA persimmon “*Diospyros kaki* Thunb. Nishimurawase” (NM), and “*Diospyros kaki* Thunb. Taifu” (TF), using the rootstock “*Diospyros Lotus* Linn” (DL) as the control, to conduct the integrated metabolomic-transcriptomic analysis to investigate the flavonoid biosynthesis, mineral absorption, and degradation of aromatic compounds from their leaves, so as to estimate and recommend the suitable persimmon leaves for making persimmon leaf tea with higher and specified functional benefits.

In this study, flavonoid biosynthesis and isoflavonoid biosynthesis from the three compared groups of DL vs. HS, DL vs. NM, and DL vs. TF were top enriched in the KEGG terms of the differentially accumulated metabolites (DAMs; Figures 2D–F) and 229 common metabolites were obtained (Figure 2G). Meanwhile, the KEGG pathway of flavonoid biosynthesis was top enriched in the three comparisons and the KEGG pathway of isoflavonoid biosynthesis was top enriched in the comparison of DL vs. HS (Figure 7A). In detail, 30, 37, and 34 DEGs related to flavonoid biosynthesis and 6, 6, and 3 DEGs related to isoflavonoid biosynthesis were obtained from the comparison of DL vs. HS, DL vs. NM, and DL vs. TF, respectively (Figure 7B). Of the above DEGs, 2 DEGs (*DN44356_c5_g1* and *DN46460_c0_g2*) related to flavonoid biosynthesis and 4 DEGs (*DN63124_c2_g2*, *DN27169_c0_g1*, *DN63124_c2_g1*, and *DN50820_c3_g2*) related to isoflavonoid biosynthesis simultaneously met with the parameters of | log2(fold change)| ≥ 1 and *P*-value < 0.05 in the three comparisons, with log2(fold change) of 3.44–8.57 (Figure 8B), which were, thus, selected for the following qRT-PCR validation. Fortunately, qRT-PCR validation showed that the relative expression level of these 6 DEGs exhibited consistent trends with the FPKM of each DEG from RNA-seq data, respectively, with log2(fold change) of 0.49–15.79 (Figure 8D).

Mineral elements of food are stable and can be used as the signature of geographical origin of the product (Zhao et al., 2017b; Li et al., 2018). The mineral contents and the quality of tea leaves were influenced by the factors of geographical origin, variety, climate conditions, harvest season, or growth stage (Jia et al., 2016; Zhao et al., 2017a). Among the factors, species and variety/cultivar played the important role in the mineral contents and the tea quality of the plants, which were grown in the geographic location.

In this study, mineral absorption from the three compared groups of DL vs. HS, DL vs. NM, and DL vs. TF was not top enriched in the KEGG terms of the DAMs (Figures 2D–F) and the KEGG pathway of mineral absorption was also not top enriched in the transcriptome profiles from the three comparisons (Figure 7A). However, 9, 12, and 12 DEGs related to mineral absorption were obtained from the comparison of DL vs. HS, DL vs. NM, and DL vs. TF, respectively (Figure 7B). Of which, 6 DEGs, including 2 downregulated DEGs (*DN41367_c2_g1* and *DN25744_c0_g1*) and 4 upregulated DEGs (*DN35062_c3_g1*, *DN45693_c0_g2*, *DN51444_c1_g2*, and *DN51444_c1_g1*) simultaneously met with the parameters of | log2(fold change)| ≥ 1 and *P*-value < 0.05 in the three comparisons, with | log2(fold change)| of 1.34–6.15 (Figure 8B), which were then selected for the following qRT-PCR validation. The qRT-PCR validation showed that the relative expression level of the 6 DEGs exhibited consistent trends with the FPKM of each DEG from RNA-seq data, respectively (2 downregulated and 4 upregulated), with | log2(fold change)| of 0.26–10.21 (Figure 8D).

As a functional beverage, persimmon leaf tea attracts more and more consumers partly due to the unique flavors, especially the aroma contributed by volatile aroma compounds. Aroma compounds come from aroma compound precursors, such as aromatic compounds, through several different pathways by endoenzymatic reaction during manufacturing processes (Feng et al., 2019; Guo et al., 2019; Amorim et al., 2020). The potential level in degradation of aromatic compounds played an important part in the generation of tea flavors and the number of volatile aroma compounds is diverse in persimmon types and their varieties (Wang et al., 2012). In this study, degradation of aromatic compounds from the three compared groups of DL vs. HS, DL vs. NM, and DL vs. TF was not top enriched in the KEGG terms of the DAMs (Figures 2D–F) and the KEGG pathway of degradation of aromatic compounds was also not top enriched in the transcriptome profiles from the three comparisons (Figure 7A). However, 3, 4, and 3 DEGs related to the degradation of aromatic compounds were obtained from the comparison of DL vs. HS, DL vs. NM, and DL vs. TF, respectively (Figure 7B) and the 3 common DEGs met simultaneously with the parameters of | log2(fold change)| ≥ 1 and *P*-value < 0.05 in the three comparisons, with log2(fold change) of 1.16–4.16 (Figure 8B); thus, the 3 DEGs were selected for the following qRT-PCR validation. Similar to mineral absorption, flavonoid biosynthesis, and isoflavonoid biosynthesis, the relative expression level of the 3 DEGs exhibited consistent trends with the FPKM of each DEG from RNA-seq data, respectively, with log2(fold change) of 1.20–8.75 (Figure 8D).

In this study, of the 6 DEGs involved in flavonoid biosynthesis, which will determine the level of total flavonoid in persimmon leaves, the | log2(fold change)| of the FPKM of 4 DEGs (*DN44356_c5_g1*, *DN46460_c0_g2*, *DN63124_c2_g2*, and *DN50820_c3_g2*), 1 DEG (*DN27169_c0_g1*), and 1 DEG (*DN63124_c2_g1*) from comparisons of DL vs. NM was significantly greater than, significantly less than, and had no significant difference with those from a comparison of DL vs. HS. For the comparison of DL vs. TF, the | log2(fold change)| of the FPKM of 3 DEGs (*DN44356_c5_g1*, *DN46460_c0_g2*, and *DN50820_c3_g2*) and 3 DEGs (*DN63124_c2_g2*, *DN27169_c0_g1*, and *DN63124_c2_g1*) was significantly greater and significantly less than those from comparison of DL vs. HS, respectively (Figure 8B), i.e., the expression level of flavonoid biosynthesis from the leaves of *Diospyros kaki* Thunb. Nishimurawase (NM) was significantly higher than *Diospyros kaki.* Heishi (HS) and the flavonoid biosynthesis from the leaves of *Diospyros kaki* Thunb. Taifu (TF) was at least at an equivalent level with HS. These results represented a greater level of total flavonoid in the NM leaves (PCNA persimmon) than TF (PCNA persimmon) and HS (PCA persimmon) leaves and equivalent level of total flavonoid in TF and HS leaves, which were a little different from the report by Chang et al. (2019), in which, the leaves from PCA persimmons had higher levels of total flavonoid and leaves from PVNA persimmons had lower levels of total flavonoid. Therefore, it could be deduced that the leaves from the same type of persimmons might have different levels of total flavonoid, while the leaves from the different types of persimmons would have similar levels of total flavonoid.

According to the mineral contents in persimmon, Tardugno et al. (2021) evaluated the mineral composition of persimmon fruits from different regions; the results showed that the mineral profile of persimmon fruits was variable across the regions. But up to now, there were no reports about the mineral contents or composition from different varieties/cultivars of persimmons grown in the same region. In this study, of the 6 DEGs involved in mineral absorption, which will determine the mineral contents from the persimmon leaves, the | log2(fold change)| of 1 DEG (*DN25744_c0_g1*), 4 DEGs (*DN41367_c2_g1*, *DN35062_c3_g1*, *DN51444_c1_g2*, and *DN51444_c1_g1*), and 1 DEG (*DN45693_c0_g2*) from a comparison of DL vs. NM was significantly greater than, significantly less than, and have no significant difference with those from a comparison of DL vs. HS, respectively; the | log2(fold change)| of 2 DEGs (*DN25744_c0_g1* and *DN45693_c0_g2*), 3 DEGs (*DN41367_c2_g1*, *DN35062_c3_g1*, and *DN51444_c1_g1*), and 1 DEG (*DN51444_c1_g2*) from a comparison of DL vs. TF was significantly greater than, significantly less than, and have no significant difference with those from a comparison of DL vs. HS, respectively (Figure 8B). In a word, the expression level of mineral absorption from the leaves of NM and TF was both less than that of HS, i.e., the leaves of HS had a greater level of mineral contents than those of NM and TF.

According to the flavor compounds in persimmon, Wang et al. (2022) reported the influence of microbial diversity on the flavor metabolites during the persimmon vinegar fermentation. However, there were no reports about the influence of persimmon variety/cultivar on the flavor metabolites and their contents. In this study, of the 3 DEGs involved in the degradation of aromatic compounds, which will determine the level of flavor metabolites, especially aroma compounds in persimmon leaves, the | log2(fold change)| of 1 DEG (*DN62817_c3_g3*), 1 DEG (*DN35893_c0_g1*), and 1 DEG (*DN62817_c3_g2*) from the comparison of DL vs. NM was significantly greater than, significantly less than, and have no significant difference with those from the comparison of DL vs. HS, respectively; the | log2(fold change)| of all the 3 DEGs from the comparison of DL vs. TF was significantly greater than those from the comparison of DL vs. HS, respectively (Figure 8B). In other words, the degradation of aromatic compounds from the leaves of NM was at least at an equivalent level with HS and the degradation of aromatic compounds from the leaves of TF was significantly greater than HS.

The following qRT-PCR validation showed that the relative expression level of all these 15 DEGs exhibited consistent trends with the FPKM of each DEG from RNA-seq data; in addition, the conjoint analysis confirmed the positive correlation between the DEGs and their metabolites; these results indicated the reliability of the integrated metabolomic-transcriptomic analysis in this study.

In conclusion, the leaves of *Diospyros kaki* Thunb. Nishimurawase (NM) had a greater level of flavonoid biosynthesis and less level of mineral absorption than those of *Diospyros kaki.* Heishi (HS) and had an equivalent level in degradation of aromatic compounds with HS; the leaves of *Diospyros kaki* Thunb. Taifu (TF) had an equivalent level with those of *Diospyros kaki.* Heishi (HS) and had a less level in mineral absorption and a significantly greater level in degradation of aromatic compounds than *Diospyros kaki.* Heishi (HS), respectively, i.e., of the three persimmon varieties in this study, the leaves of *Diospyros kaki* Thunb. Nishimurawase (NM) had the greatest level in flavonoid biosynthesis, in the leaves of *Diospyros kaki.* Heishi (HS) had the greatest level in mineral absorption, and the leaves of *Diospyros kaki* Thunb. Taifu (TF) had the greatest level in degradation of aromatic compounds. Thus, the tender leaves from *Diospyros kaki.* Heishi (HS), *Diospyros kaki* Thunb. Nishimurawase (NM), and *Diospyros kaki* Thunb. Taifu (TF) could be recommended for the production of persimmon leaf tea rich in mineral elements, total flavonoid, and aroma compounds, respectively.

## Data Availability Statement

The original contributions presented in the study are included in the article/supplementary material, further inquiries can be directed to the corresponding author.

## Author Contributions

X-MY and C-XA designed the experiments. X-MY performed most of the experiments, analyzed the data, and wrote the manuscript. All authors assisted in experiments, discussed the results, and commented on the final version of the manuscript.

## Conflict of Interest

The authors declare that the research was conducted in the absence of any commercial or financial relationships that could be construed as a potential conflict of interest.

## Publisher’s Note

All claims expressed in this article are solely those of the authors and do not necessarily represent those of their affiliated organizations, or those of the publisher, the editors and the reviewers. Any product that may be evaluated in this article, or claim that may be made by its manufacturer, is not guaranteed or endorsed by the publisher.

## Footnotes

1
www.metware.cn

2
http://www.bioinformatics.babraham.ac.uk/projects/fastqc/

3
https://github.com/trinityrnaseq/trinityrnaseq/wiki

4
https://www.ncbi.nlm.nih.gov/search/all/?term=PRJNA646845
